# Integrating Next Generation sequencing into the risk assessment of primary myelofibrosis

**DOI:** 10.1016/j.htct.2026.106532

**Published:** 2026-09-24

**Authors:** Rafaela Reis-Vieira, Juliana Bulchi da Costa, Diego Coutinho, Leonardo Javier Arcuri, Mariana D'Andrea, Maria Laura Robledo, Adelmo H. Daumas, Cristiana Solza, Mariana Boroni, Ilana Zalcberg, Barbara da Costa Reis Monte-Mór

**Affiliations:** a Instituto Nacional de Câncer - Brazilian National Cancer Institute, Rio de Janeiro, Brazil; b Hospital Universitário Antônio Pedro - Universidade Federal Fluminense, Niterói, Rio de Janeiro, Brazil; c Hospital Universitário Pedro Ernesto - Universidade do Estado do Rio de Janeiro, Rio de Janeiro, Brazil

To the Editor

Primary myelofibrosis (PMF) is a myeloproliferative neoplasm (MPN) driven by activating mutations in the JAK/STAT pathway. The *JAK2* V617F mutation is detected in approximately 60% of patients, *CALR* indels in 25–30%, and *MPL* mutations in 5–10%. A subset of patients lacks disease driver mutations and is classified as triple-negative [1]. The mutational landscape of PMF includes other clonal driver mutations affecting genes involved in epigenetic regulation, mRNA splicing, transcriptional regulation, and cell-signaling pathways. Disease-driver and clonal-driver mutations can influence disease presentation and clinical outcomes in patients with PMF, and genomic data are increasingly incorporated into prognostic scores [[2], [3], [4], [5]]. Here, we used a customized target Next Generation Sequencing (NGS) panel to detect mutations in myeloid driver genes in a retrospective real-world series of patients with PMF treated within the public health system in Rio de Janeiro, Brazil. This study aimed to use genomic information to stratify risk and examine its association with disease outcomes.

A total of 32 patients were included, with a median age of 62.5 years at the time of PMF diagnosis (Table S1). Twelve patients (37.5%) had hemoglobin <10 g/dL, 17 (53.1%) had peripheral blood blasts ≥1%, 21 (65.6%) had splenomegaly, and three had a history of thrombosis. At least one constitutional symptom was observed in 12 patients. According to the International Prognostic Scoring System (IPSS), 22% of patients were classified as low risk, 25% as Intermediate-1, 25% as Intermediate-2, and 28% as high risk. Median follow-up time from diagnosis was 5.04 years (95% CI: 2.77–6.73).

Disease-driver mutations were screened using single-gene approaches, and myeloid clonal driver mutations were detected using NGS (Supplemental material). Among the 32 patients, 14 (43.7%) had DNA samples collected within three months of diagnosis, and the median time from diagnosis to DNA sampling was 7.2 months (Figure S1). A total of 82 mutations were identified across 20 genes (Figure 1 and Table S2). The number of mutations per patient ranged from one to seven, with 22% exhibiting four or more mutations. No significant correlation was found between the number of mutations and the timing of DNA sampling (r = −0.004, p = 0.98). Indeed, clonal evolution in chronic-phase MPN is typically slow, as shown by phylogenetic mutational studies and longitudinal sampling after diagnosis [2,6].

**Figure 1:**
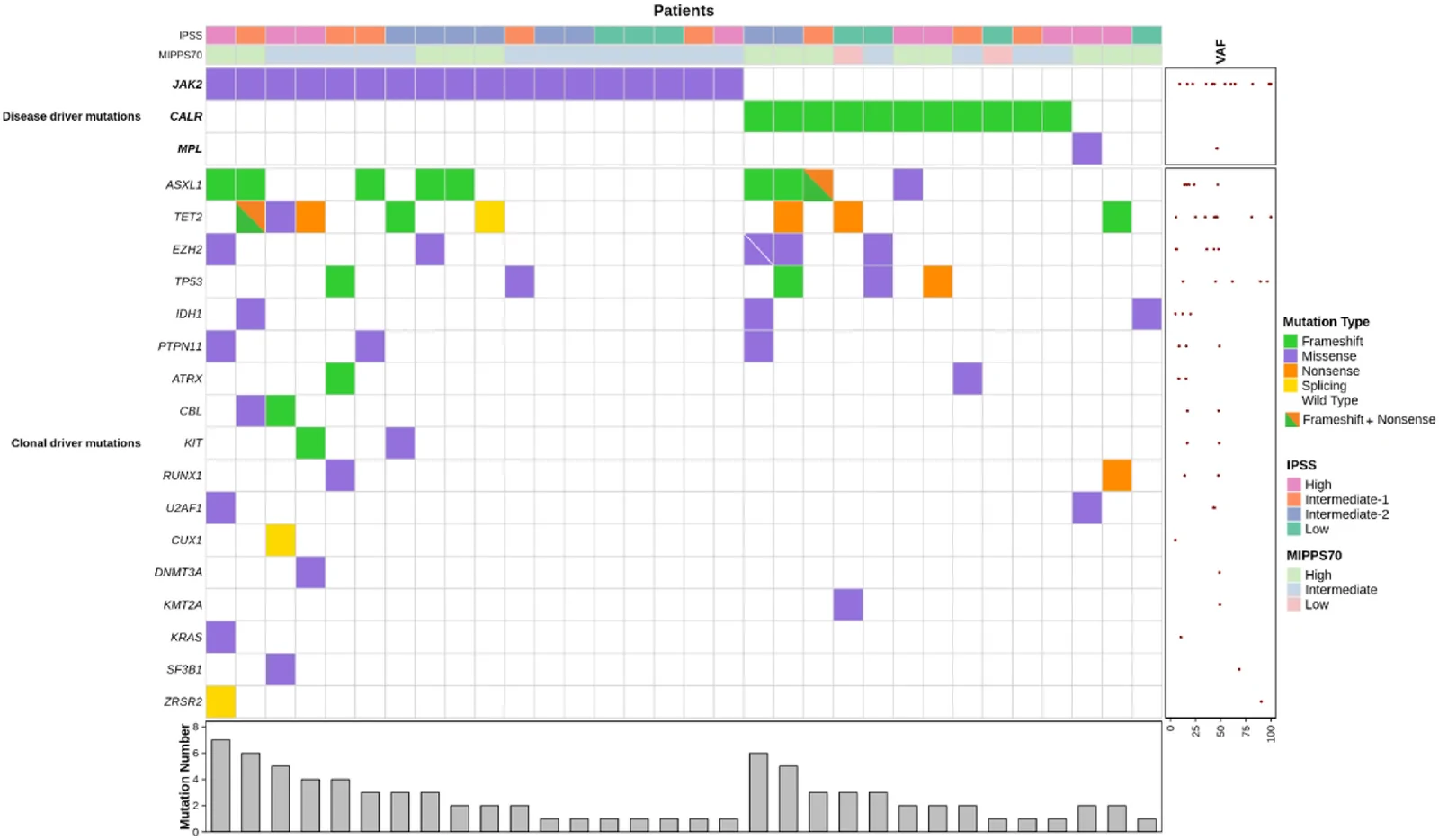
Molecular profile and overall survival in primary myelofibrosis.

Disease-driver mutations were analyzed using single-gene approaches, and other clonal driver mutations were screened using a custom Next Generation Sequencing myeloid gene panel. Each column represents a patient, and each row a gene. In the right panel, circles represent the variant allele frequency (VAF). The total number of mutations per patient is shown in the lower panel. Different colors represent mutation types (frameshift, missense, nonsense, or affecting a splice site).

A driver mutation was detected in 94% of patients: *JAK2* V617F in 54%, *CALR* indels in 36% (type-1: 24%, type-2: 12%), and *MPL* W515L in one. Two patients were triple-negative. *JAK2* V617F, compared to *CALR* indels, was associated with higher age, hemoglobin levels, and white blood cell counts (Table S3). Approximately 70% of patients harbored other clonal driver mutations, predominantly frameshift and nonsense mutations affecting *ASXL1* and *TET2,* present in 28% and 25% of patients, respectively. A novel 22 bp deletion in *ASXL1* identified by NGS was confirmed as somatic (Figure S2). At least one high-molecular risk (HMR) mutation (*ASXL1, EZH2, SRSF2, IDH1/2*) [3] was detected in 34% of patients, with five patients presenting two or more HMR mutations. *TP53* mutations were found in five patients. Overall, the somatic mutational landscape in our PMF series resembles that reported in larger cohorts [2,4,7].

We used mutational and clinical data to assess patient risk according to the Mutation Enhanced International Prognostic Score System (MIPSS70) [5]. MIPSS70 was originally designed for transplant-age patients, and 29 of 32 patients in our series were 70 years old or younger. As MIPSS70 has also demonstrated prognostic utility in all-age PMF cohorts, it was applied to all patients in this study. Compared with IPSS, 37.5% of patients were reclassified into higher-risk categories by MIPSS70. This included 5 of 7 IPPS low-risk patients, and 7 of 16 IPPS intermediate-1/2 patients (Figure 2A). Patients who were upstaged lacked *CALR* type 1 mutations or harbored HMR mutations or both. Conversely, only 16% of patients were downstaged: 5 of 9 IPSS high-risk patients were reclassified into the MIPSS70 intermediate-risk category. Moreover, we applied the Sanger MPN tool to assess personalized prognosis for each patient [4]. Predicted event free survival ranged from 2–22 years (Figure 2B). These results underscore the importance of integrating NGS molecular analysis to improve risk assessment in a real-world setting.

**Figure 2:**
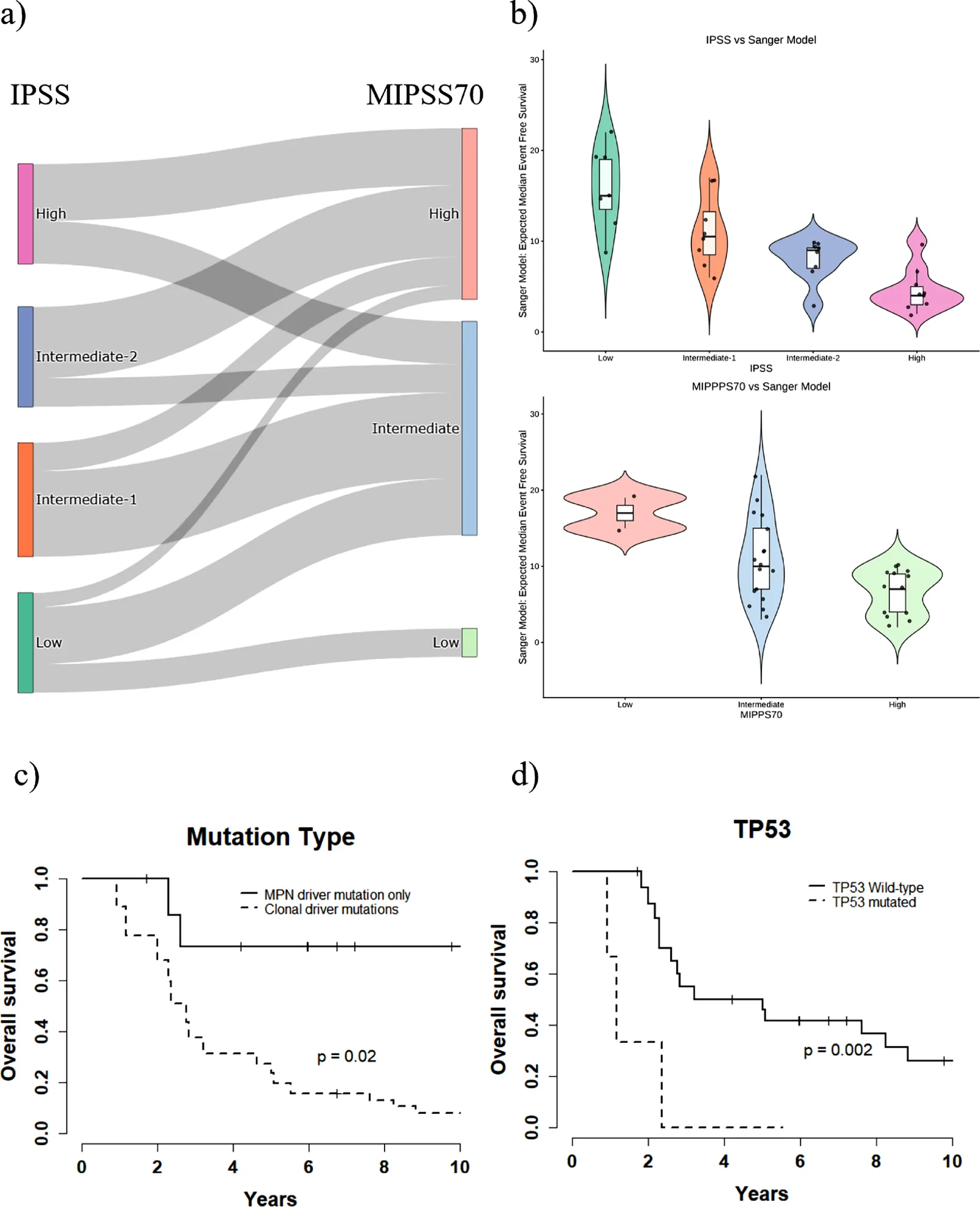
Risk assessment and overall survival in primary myelofibrosis.

**a)** Sankey plot showing risk classification according to International Prognostic Scoring System (right) and Mutation Enhanced International Prognostic Score System (MIPSS70) (left); **b)** Individual event free survival calculated using to Sanger model, distributed according to IPPS (upper plot) or MIPSS70 (lower plot); **c)** and **d)** Kaplan–Meier estimates of overall survival (late-entry Cox model) according to **c)** the presence or absence of at least one myeloid clonal driver mutation; p = 0.02; or **d)***TP53* mutational status.

The median overall survival (OS) from diagnosis for all patients was 5.52 years (95% CI: 2.82 to not reached). Because the DNA samples available for NGS were not always from the time of diagnosis, to evaluate the impact of genomic variables and account for potential immortal time bias, all subsequent analyses were calculated from the time of DNA sampling. The median OS from DNA sampling for all patients was 2.82 years (95% CI: 2.29–8.24 years). The number of mutations per patient impacts the OS (Figure S3), and the presence of at least one clonal-driver mutation was associated with shorter OS (Figure 2C). In our series, in contrast to larger studies, no significant difference in survival was observed according to HRM mutation status. This result may be limited by sample size (Figure S3) [3]. While *EZH2* mutations were associated with reduced OS, *ASXL1* mutations did not significantly affect it (Figure S3). *ASXL1* mutations have been associated with worse prognosis in PMF [3]. In contrast to prior reports, the study by Luque Paz et al. found that these mutations alone, in the absence of *TP53* or other HRM mutations, did not retain prognostic value [8]. In this report, we found that *TP53* mutations were associated with reduced OS (Figure 2D). Indeed, *TP53* mutations have been correlated with poorer outcomes and a higher risk of leukemic transformation in PMF [2,4,[8], [9], [10], [11]]. *TP53* mutations are often detected at low variant allele frequencies (VAFs) at the time of MPN diagnosis, but clonal evolution can occur as the disease progresses. Leukemic transformation is associated with loss of the second allele and a VAF >50% [2]. Recent data reinforce the prognostic relevance of *TP53* multihit mutations in chronic phase myelofibrosis and suggest it should be included in the group of ‘myeloid neoplasms with mutated *TP53*’ of the International Consensus Classification [10,11]. In our series of PMF patients, 3 of 5 *TP53* mutations had VAF >50%. Although this report is based on a retrospective analysis of a small and heterogeneous subset of patients, our data also suggest an adverse impact of *TP53* mutations on disease outcome, supporting their inclusion in PMF prognostic scores.

Access to NGS mutational analysis is increasing; however, it is still not widely available in Brazil, particularly within the public health system. Our study demonstrates that molecular profiling offers valuable insights and should be incorporated into the clinical management of patients with PMF.

## Ethical approval statement

The study was approved by INCA’s Research Ethics Committee and registered at Plataforma Brasil (CAAE 65,974,117.9. 0000.5274). All patients signed an informed consent form.

## Authorship contributions

BMM conceived and designed the study, performed experiments, interpreted data, and wrote the manuscript. IZ conceived and designed the study, interpreted data and contributed to the paper's writing. RRV performed experiments, interpreted the data, prepared figures, and wrote the paper. AHD and CS made the diagnoses, sent patients’ samples, collected clinical and laboratory data, and revised the manuscript. DC, MLR, and MDA performed experiments. MB analyzed genomic data. LJA and JBC analyzed the data, prepared the figures, and contributed to the writing. All authors read and approved the final manuscript.

## Conflicts of interest

IZ is a consultant to DASA Genomics. CS has received speaker honorarium and served on the advisory board for Novartis Oncology. All other authors have no conflict of interest to disclose.

## Data Availability

All data are available within the text.

## Appendix Supplementary materials

Image, application 1
