# Supplementary material for: Integrating Next Generation sequencing into the risk assessment of primary myelofibrosis
Source: Hematol Transfus Cell Ther. 2026 Sep 24;48(4):106532. doi: 10.1016/j.htct.2026.106532 (PMC13634431; doi:10.1016/j.htct.2026.106532)
Supplement: Supplementary file 1 [file mmc1.docx]

**Supplemental information**

**Integrating Next Generation Sequencing into the risk assessment of primary myelofibrosis**

**Supplemental methods**

**Patients and samples**

From 2008 to 2014, 2124 samples from 1452 patients from hematology services in Rio de Janeiro were referred to the Molecular Biology Laboratory at Instituto Nacional de Câncer (INCA) for *JAK2* V617F analysis at diagnosis or follow-up. Granulocytes were isolated using a Ficoll-Histopaque (Sigma-Aldrich) density gradient, and genomic DNA was extracted using the automated Maxwell system (Promega).

Next Generation Sequencing (NGS) was performed on thirty-two patients from two university hospitals in Rio de Janeiro state, Pedro Ernesto (HUPE/UERJ) and Antônio Pedro (HUAP/UFF), with a confirmed diagnosis of primary myelofibrosis (PMF) who had available clinical data and stored DNA samples. Patients diagnosed with secondary myelofibrosis, limited clinical information, or low-quality DNA samples that hindered NGS performance or analysis were excluded. The Myeloproliferative Neoplasm Symptom Assessment Form - Total Symptom Score (MPN-SAF TSS) was used to assess constitutional symptoms [1]. Follow-up information was obtained until December 2019. The study was approved by the Research Ethics Committee of INCA (CAAE 65974117.9.0000.5274), and patients signed an informed consent.

**Detection of myeloproliferative neoplasm** **driver mutations**

As part of the diagnostic workup, DNA samples from PMF patients were first screened for the *JAK2* V617F mutation by allele-specific Polymerase Chain Reaction (PCR) [2]. *JAK2* V617F-negative samples were further analyzed for insertions and deletions (indels) in *CALR* exon 9 by PCR using fluorescently labeled primers, followed by fragment analysis on the 3130xl gene analyzer (Applied Biosystems) [3]. *JAK2/CALR* double-negative samples were then analyzed for *MPL* exon 10 mutations by PCR followed by direct Sanger sequencing. All primers used are presented in Table S4.

**Next-generation sequencing and direct sequencing**

NGS was used to detect exon-coding and splicing site mutations in a custom panel of 37 genes recurrently mutated in myeloid neoplasms (*AKT1, ASXL1, ATRX, BAP1, BCOR, CBL, CBLB, CEBPA, CUX1, DNMT3A, ETV6, EZH2, FLT3, HRAS, IDH1, IDH2, JAK2, KIT, KRAS, MLL, MPL, NOTCH1, NPM1, NRAS, PHF6, PTEN, PTPN11, RUNX1, SF3B1, SRSF2, SUZ12, TET2, TP53, U2AF1, UTX, WT1*, and *ZRSR2*) [4]. Genomic libraries were prepared with the Ion AmpliSeq™ Library Kit 2.0, emulsion PCR was performed on the Ion OneTouch™ using the Ion PGM™ Hi-Q™ OT2 Kit, and sequencing was carried out on the Ion Personal Genome Machine ™ using the Ion PGM™ Hi-Q™ Sequencing Kit (PGM, Thermo Fisher Scientific, MA, USA). Five hundred and eighty-eight exons (1099 amplicons) were sequenced, corresponding to >109 Kb per sample.

Mutation analysis was performed using the Ion Reporter Software (Thermo Fisher Scientific) for variant calling and annotation. To enrich for clinically relevant mutations, we filtered out variants: 1) located in untranslated regions (UTRs) or deeply in introns; 2) annotated as synonymous; 3) annotated as missense present in the dbSNP database (rs) and absent in COSMIC; 4) annotated as missense and scored as benign by *in silico* tools SIFT and POLYPHEN; or 5) with a depth of coverage (DP) <30, for variants not described in dbSNP or COSMIC databases.

In addition, to further evaluate single nucleotide variant (SNV) calling and annotation, the GATK best practices pipeline (Broad Institute Cambridge, MA, USA) was used. Variants were annotated using the Ensembl Variant Effect Predictor (VEP). Briefly, variants were filtered out when: 1) DP <6; 2) global minor allele frequency (GMAF) >0.01 based on 1000 Genome and dbSNP databases; 3) predicted as LOW (synonymous) or MODIFIER (translated regions - UTRs; introns); or 4) classified as benign by both SHIFT and POLYPHEN.

Variants were curated and subjected to visual inspection using the Integrative Genomics Viewer (IGV) software to obtain a final list of mutations in our PMF cohort. Variants were filtered out when located: 1) at the end of an amplicon; 2) in homopolymeric regions (for variants not described in the COSMIC database); or 3) in only one sequencing sense.

Mutations of the two most frequently affected genes in our cohort, *TET2* and *ASXL1,* were confirmed by PCR followed by direct Sanger sequencing using BigDye™ Terminator v3.1 Cycle Sequencing Kit (Thermo Fisher Scientific) (Table S4).

**Sanger multistage model of myeloproliferative neoplasm** **personalized risk assessment**

The online tool developed by Grinfeld et al. (https://cancer.sanger.ac.uk/mpn-multistage/) was used to assess individual event-free survival predictions for each PMF patient studied [5]. An advantage of this multistage, multifactorial Bayesian model and the online platform is that it can be performed even if some variables are missing. Variables available in our PMF patient cohort were incorporated into the risk calculation: demographic (gender and age, at diagnosis or at DNA sampling when appropriate), clinical and laboratory (thrombosis, splenomegaly, hemoglobin concentration, leukocyte, and platelet count), and genomics (presence or absence of mutations in 23 genes: *JAK2* V617F or exon 12, *CALR, MPL* [S204/S505/W515 hotpots], *ASXL1, BCOR, CBL, CUX1, DNMT3A, EZH2, IDH1, IDH2, KIT, KRAS, NRAS, PHF6, PTPN11, RUNX1, SF3B1, SRSF2, TET2, TP53, U2AF1*, and *ZRSR2*). Variables not available were set as ‘unknown’.

**Statistical Analysis**

The ‘survival’ package analyzed demographic, laboratory, and clinical data using R (Foundation for Statistical Computing, Vienna, Austria), version 4.1.0. Profiles of the patients were presented as numbers and percentages, mean and standard deviation (SD), or median and range, as appropriate. Outcomes were presented as numbers and rates. Survival curves were built with the Kaplan-Meier method for late entry. Comparisons were performed in late-entry Cox models. In late-entry models, patients are clinically diagnosed at T0 but only enter the analysis cohort at T1 when the specimen is sent for genetic analysis. They stay in the analysis cohort until T2, when outcome or censoring occurs.

**References**

1. Guaraná M, Soares A, Daumas A, Biasoli I, Solza C Myeloproliferative Neoplasm Symptom Assessment Form - Total Symptom Score (MPN-SAF TSS) questionnaire: translation, cultural adaptation and validation to Brazilian Portuguese. Hematology, Transfusion and Cell Therapy 2022 44(3):321-7. https://doi.org/10.1016/j.htct.2020.10.966
2. Baxter EJ, Scott LM, Campbell PJ, East C, Fourouclas N, Swanton S, et al. (2005) Acquired mutation of the tyrosine kinase JAK2 in human myeloproliferative disorders. Lancet 365(9464):1054-61. https://doi.org/10.1016/S0140-6736(05)71142-9
3. Klampfl T, Gisslinger H, Harutyunyan AS, Nivarthi H, Rumi E, Milosevic JD, Them NC, Berg T, Gisslinger B, Pietra D, Chen D, Vladimer GI, Bagienski K, Milanesi C, Casetti IC, Sant'Antonio E, Ferretti V, Elena C, Schischlik F, Cleary C, Six M, Schalling M, Schönegger A, Bock C, Malcovati L, Pascutto C, Superti-Furga G, Cazzola M, Kralovics R (2013) Somatic mutations of calreticulin in myeloproliferative neoplasms. N Engl J Med 369(25):2379-90. https://doi.org/10.1056/NEJMoa1311347
4. Coutinho DF, Boroni M, Batalha ABW, et al. Somatic genomic variants in refractory cytopenia of childhood. Pediatric Hematology Oncology Journal 2021 6(3):123-126. https://doi.org/10.1016/j.phoj.2021.04.180
5. Grinfeld J, Nangalia J, Baxter EJ, et al. Classification and Personalized Prognosis in Myeloproliferative Neoplasms. N. Engl. J. Med. 2018 379(15): 1416–1430. https://doi.org/10.1056/NEJMoa1716614

**Supplemental figures**

**Figure S1:** Follow-up time of primary myelofibrosis (PMF) patients

The 32 PMF patients included in the study are represented on the X-axis, and follow-up (days) is on the Y-axis. The dashed line represents the time between diagnosis and DNA sampling.

**Patients #**

**Days**

**Figure S2:** Validation of *TET2* and *ASXL1* mutations by Sanger sequencing

**a)** Electropherograms showing, in the left panel, mutations identified in *TET2 (*c.3954+1G>A; c.3812dup and c.3646C>T), and in the right panels, mutations in *ASXL1 (*c.1934dup; c.2385del and c.2077C>T); **b)** Electropherograms showing, in the left panel, a polymorphism in *ASXL1* (c.1941G>A) identified in granulocytes (Gr) and CD3+ cells of a PMF patient (#26). For the same patient, a new 22 bp deletion (c.2134_2155delGCCATGTCCAGAGCTAGGAGAG) in *ASXL1* was confirmed as somatic, as shown in the right panel (positive in Gr, negative in CD3+ cells).

**Figure S3:** Overall survival (OS) in a primary myelofibrosis (PMF) series

**a)** Kaplan–Meier curve of OS for 32 patients with PMF, calculated from the time of DNA sampling. The horizontal axis represents the survival time (in years). The dashed lines represent the 95% confidence interval. **b-e)** Kaplan–Meier curve of overall survival probability according to the number of mutations (b), High molecular risk mutations status (c), *EZH2* mutational status (d), and *ASXL1* mutational status (e), all calculated from the time of DNA sampling.

| **Table S1: Clinical, demographic, and laboratory features** | |
| --- | --- |
| PMF patients in the study - n | 32 |
| At diagnosis |  |
| Age - median (range) | 62.5 years (42-77) |
| Gender - n (%) | M: 18 (56%) / F: 14 (44%) |
| Hemoglobin - median (range) | 11.0 g/dL (4.0-19.6) |
| White blood cells - median (range) | 10.8 x 10⁹/L (2.1-57.6x10⁹) |
| Platelets - median (range) | 386 x 10⁹/L (77-1435x10⁹) |
| Thrombosis - n | 3 |
| Splenomegaly - n | 21 |
| Weight loss - n | 12 |
| Fever - n | 2 |
| Night sweats - n | 1 |
| IPSS |  |
| Low - n (%) | 7 (22) |
| Intermediate-1 - n (%) | 8 (25) |
| Intermediate-2 - n (%) | 8 (25) |
| High - n (%) | 9 (28) |
| Treatment |  |
| Hydroxyurea or other cytoreductive agents - n | 23 |
| Thalidomide and/or corticosteroids - n | 9 |
| Erythropoietin - n | 3 |
| Androgens - n | 0 |
| Ruxolitinib - n | 2 |
| HCT - n | 1 |
| Splenectomy or Splenic Radiation - n | 2 |
| Follow up from diagnosis - median | 5.04 years |
| Deaths - n | 20 |
| Associated with AML transformation - n | 3 |
| PMF: primary myelofibrosis; IPSS: International Prognostic Scoring System; HCT: allogeneic hematopoietic cell transplantation; AML: acute myeloid leukemia | |

| **Table S2: Molecular features** | | | | | | | | | |
| --- | --- | --- | --- | --- | --- | --- | --- | --- | --- |
| **Disease driver mutations** |  |  |  |  |  |  |  |  |  |
| ***JAK2*** | JAK2 p.Val617Phe (18) |  |  |  |  |  |  |  |  |
| ***CALR*** | CALR p.L367fs*46 (7) | CALR p.K385fs*47 (4) |  |  |  |  |  |  |  |
| ***MPL*** | MPL p.Trp515Leu |  |  |  |  |  |  |  |  |
| **Clonal driver mutations** |  |  |  |  |  |  |  |  |  |
| ***ASXL1*** | ASXL1 p.G646fs*12 (4) | ASXL1 p.Arg693Ter (2) | ASXL1 p.Tyr591fs | ASXL1 p.Ala712fs | ASXL1 p.W796Gfs*22 | ASXL1 p.Asp832fs |  |  |  |
| ***TET2*** | TET2 splicesite_3 | TET2 p.C1271Wfs*29 | TET2 p.Arg1216Ter | TET2 p.Gly1391Asp | TET2 p.Cys1263Tyr | TET2 p.Ser1586Ter | TET2 p.Gly898Ter | TET2 p.Gln969Ter | TET2 p.Ser231fs |
| ***EZH2*** | EZH2 p.Ile650Thr | EZH2 p.Arg690His | EZH2 p.Asn673Ser | EZH2 p.Cys468Ser | EZH2 p.Arg684Cys | EZH2 p.Gly660Arg |  |  |  |
| ***TP53*** | TP53 p.Gly245Asp | TP53 p.Pro190fs | TP53 p.Trp146Ter | TP53 p.Pro152fs*18 | TP53 p.Arg273Cys |  |  |  |  |
| ***IDH1*** | IDH1 p.Arg132Cys | IDH1 p.Arg132His | IDH1 p.Ser210Asn |  |  |  |  |  |  |
| ***PTPN11*** | PTPN11 p.Gln79Arg | PTPN11 p.Arg265Gln | PTPN11 p.Asn308Asp |  |  |  |  |  |  |
| ***ATRX*** | ATRX p.Asn1694Ser | ATRX p.Arg1302fs*44 |  |  |  |  |  |  |  |
| ***CBL*** | CBL p.Asp390Val | CBL p.Ala757fs |  |  |  |  |  |  |  |
| ***KIT*** | KIT p.Leu682fs | KIT p.Asp816Asn |  |  |  |  |  |  |  |
| ***RUNX1*** | RUNX1 p.Arg201Ter | RUNX1 p.Gly135Asp |  |  |  |  |  |  |  |
| ***U2AF1*** | U2AF1 p.Gln157Pro (2) |  |  |  |  |  |  |  |  |
| ***CUX1*** | CUX1 splicesite_3 |  |  |  |  |  |  |  |  |
| ***DNMT3A*** | DNMT3A p.Tyr735Ser |  |  |  |  |  |  |  |  |
| ***KMT2A*** | KMT2A p.Ser3112Ile |  |  |  |  |  |  |  |  |
| ***KRAS*** | KRAS p.Gly12Ser |  |  |  |  |  |  |  |  |
| ***SF3B1*** | SF3B1 p.Lys666Asn |  |  |  |  |  |  |  |  |
| ***ZRSR2*** | ZRSR2 splice_acceptor_variant |  |  |  |  |  |  |  |  |

| Table S3: JAK2 V617F X *CALR* indels | | | |
| --- | --- | --- | --- |
|  | **JAK2 V617F** | ***CALR* indels** | **p-value** |
| n | 18 | 11 |  |
| Age, median (range) | 64 years (42-77) | 53 years (43-70) | **0.032** * |
| Gender, n | M: 12 / F: 6 | M: 4 / F: 7 | 0.143 ° |
| Hemoglobin, median (range) | 12,6 g/dL (4,0-19,6) | 10,6 g/dL (7,0-12,6) | **0.036** * |
| White blood cells, median (range) | 13,4 x10⁹/L (5,0-47,8) | 8,4 x10⁹/L (2,2-43,4) | **0.024** * |
| Platelets, median (range) | 435 x10⁹/L (105-1332) | 329 x10⁹/L (130-1435) | 0.868 * |
| Blood blasts, median (range) | 0% (0-6) | 0% (0-12) | 0.706 * |
| Thrombosis, n | 2 | 1 | 1.000 ° |
| Splenomegaly, n | 12 | 6 | 0.696 ° |
| Constitutional symptoms, n | 11 | 9 | 0.494 ° |
| * Wilcoxon rank-sum test; ° Fisher's exact test | | | |

| Table S4: Primers used in the study | | |
| --- | --- | --- |
| **Primer** | **Sequence** | **Amplicon size** |
| JAK2 F | 5’- ATCTATAGTCATGCTGAAAGTAGGAGAAAG- 3’ | 364pb - WT |
| JAK2V617F F | 5’- AGCATTTGGTTTTAAATTATGGAGTATATT- 3’ | 203pb - mutated |
| JAK2 R | 5’- CTGAATAGTCCTACAGTGTTTTCAGTTTCA- 3’ |  |
| CALR ex9F | FAM 5’- GGCAAGGCCCTGAGGTGT- 3’ | 260pb - WT |
| CALR ex9R | 5’- GGCCTCAGTCCAGCCCTG- 3’ |  |
| MPL ex10F | 5’- TCCTAGCCTGGATCTCCTTGG- 3’ | 186pb |
| MPL ex10R | 5’- CACAGAGCGAACCAAGAATGCC- 3’ |  |
| TET2 ex3 PCR1F | 5’- TGAACTTCCCACATTAGCTGGT- 3' | 955pb |
| TET2 ex3 PCR1R | 5’- GAAACTGTAGCACCATTAGGCATT- 3' |  |
| TET2 ex3 PCR2F | 5’- CAAAAGGCTAATGGAGAAAGACGTA- 3' | 836pb |
| TET2 ex3 PCR2R | 5’- GCAGAAAAGGAATCCTTAGTGAACA- 3' |  |
| TET2 ex3 PCR3F | 5’- GCCAGTAAACTAGCTGCAATGCTAA- 3' | 846pb |
| TET2 ex3 PCR3R | 5’- TGCCTCATTACGTTTTAGATGGG- 3' |  |
| TET2 ex3 PCR4F | 5’- GACCAATGTCAGAACACCTCAA- 3' | 867pb |
| TET2 ex3 PCR4R | 5’- TTGATTTTGAATACTGATTTTCACCA- 3' |  |
| TET2 ex3 PCR5F | 5’- TTGCAACATAAGCCTCATAAACAG- 3' | 788pb |
| TET2 ex3 PCR5R | 5’- ATTGGCCTGTGCATCTGACTAT- 3' |  |
| TET2 ex3 PCR6F | 5’- GCAACTTGCTCAGCAAAGGTACT- 3' | 781pb |
| TET2 ex3 PCR6R | 5’- TGCTGCCAGACTCAAGATTTAAAA- 3' |  |
| TET2 ex5F | 5’- CATTTCTCAGGATGTGGTCATAGAAT- 3’ | 286pb |
| TET2 ex5R | 5’- CCCAATTCTCAGGGTCAGATTTA- 3’ |  |
| TET2 ex6F | 5’- AGACTTATGTATCTTTCATCTAGCTCTGG- 3’ | 599pb |
| TET2 ex6R | 5’- ACTCTCTTCCTTTCAACCAAAGATT- 3’ |  |
| TET2 ex7F | 5’- ATGCCACAGCTTAATACAGAGTTAGAT- 3’ | 362pb |
| TET2 ex7R | 5’- TGTCATATTGTTCACTTCATCTAAGCTAAT- 3’ |  |
| TET2 ex9F | 5’- TGTCATTCCATTTTGTTTCTGGATA- 3' | 361pb |
| TET2 ex9R | 5’- AAATTACCCAGTCTTGCATATGTCTT- 3' |  |
| TET2 ex10F | 5’- CTGGATCAACTAGGCCACCAAC- 3’ | 774pb |
| TET2 ex10R | 5’- CAAAATTAACAATGTTCATTTTACAATAAGAG-3’ |  |
| TET2 ex11 PCR1F | 5’- GCTCTTATCTTTGCTTAATGGGTGTT- 3’ | 748pb |
| TET2 ex11 PCR1R | 5’- TGTACATTTGGTCTAATGGTACAACTG- 3’ |  |
| TET2 ex11 PCR2F | 5’- AATGGAAACCTATCAGTGGACAAC- 3’ | 1107pb |
| TET2 ex11 PCR2R | 5’- TATATATCTGTTGTAAGGCCCTGTGA- 3’ |  |

| ASXL1 ex12PCR1F | 5’- AGGTCAGATCACCCAGTCAGTT- 3’ | 561pb |
| --- | --- | --- |
| ASXL1 ex12PCR1R | 5’- TAGCCCATCTGTGAGTCCAACTGT- 3’ |  |
| ASXL1 ex12PCR2F | 5’- AGAGGACCTGCCTTCTCTGAGAAA- 3’ | 558pb |
| ASXL1 ex12PCR2R | 5’- TTCGATGGGATGGGTATCCAATGC- 3’ |  |
| ASXL1 ex12PCR3F | 5’- ACTTGAAAACCAAGGCTCTCGT- 3’ | 532pb |
| ASXL1 ex12PCR3R | 5’- GCAACCATCCCATCTGTCCTTGTA- 3’ |  |
| ASXL1 ex12PCR4F | 5’- GGTGGACAAGGATGAGAAACCCAA- 3’ | 674pb |
| ASXL1 ex12PCR4R | 5’- TGTCCTGTGACATAGCACGGACTT- 3’ |  |
| ASXL1 ex12PCR5F | 5’- TGGATTCCAAAGAGCAGTTCTCTTC- 3’ | 533pb |
| ASXL1 ex12PCR5R | 5’- CATGACAAAGGGCATCCCTTCCAA- 3’ |  |
| ASXL1 ex12PCR6F | 5’- ACAGGAAAGCTACTGGGCATAGTC- 3’ | 593pb |
| ASXL1 ex12PCR6R | 5’- CAAGAGTGCTCCTGCCTAAAGAGT- 3’ |  |
